# p16(Ink4a) and senescence-associated β-galactosidase can be induced in macrophages as part of a reversible response to physiological stimuli

**DOI:** 10.18632/aging.101268

**Published:** 2017-08-02

**Authors:** Brandon M. Hall, Vitaly Balan, Anatoli S. Gleiberman, Evguenia Strom, Peter Krasnov, Lauren P. Virtuoso, Elena Rydkina, Slavoljub Vujcic, Karina Balan, Ilya I. Gitlin, Katerina I. Leonova, Camila R. Consiglio, Sandra O. Gollnick, Olga B. Chernova, Andrei V. Gudkov

**Affiliations:** ^1^ Everon Biosciences, Inc., Buffalo, NY 14203, USA; ^2^ Department of Cell Stress Biology, Roswell Park Cancer Institute, Buffalo, NY 14263, USA; ^3^ Department of Tumor Immunology, Roswell Park Cancer Institute, Buffalo, NY 14263, USA

**Keywords:** aging, macrophage, senescent cell, p16(Ink4a), beta-galactosidase

## Abstract

Constitutive *p16^Ink4a^* expression, along with senescence-associated β-galactosidase (SAβG), are commonly accepted biomarkers of senescent cells (SCs). Recent reports attributed improvement of the healthspan of aged mice following *p16^Ink4a^*-positive cell killing to the eradication of accumulated SCs. However, detection of *p16^Ink4a^*/SAβG-positive macrophages in the adipose tissue of old mice and in the peritoneal cavity of young animals following injection of alginate-encapsulated SCs has raised concerns about the exclusivity of these markers for SCs. Here we report that expression of *p16^Ink4a^* and SAβG in macrophages is acquired as part of a physiological response to immune stimuli rather than through senescence, consistent with reports that p16^Ink4a^ plays a role in macrophage polarization and response. Unlike SCs, *p16^Ink4a^*/SAβG-positive macrophages can be induced in p53-null mice. Macrophages, but not mesenchymal SCs, lose both markers in response to M1- [LPS, IFN-α, Poly(I:C)] and increase their expression in response to M2-inducing stimuli (IL-4, IL-13). Moreover, interferon-inducing agent Poly(I:C) dramatically reduced *p16^Ink4a^* expression *in vivo* in our alginate bead model and in the adipose tissue of aged mice. These observations suggest that the antiaging effects following eradication of *p16^Ink4a^*-positive cells may not be solely attributed to SCs but also to non-senescent *p16^Ink4a^*/SAβG-positive macrophages.

## INTRODUCTION

Senescence is a cellular phenotype, initially described in cell culture, that is acquired in a variety of normal and tumor-derived cells following activation of the p53/p21^Cip1/Waf1^-, Rb/p16^Ink4a^-dependent DNA damage response and characterized by irreversible proliferation arrest coupled with a constitutive pro-inflammatory secretory phenotype (SASP) [1–3]. Accumulation of senescent cells (SCs) in mammalian tissues with age and the proinflammatory activity resulting from SASP has been proposed as a major factor responsible for aging-associated chronic systemic sterile inflammation (“inflammaging”, see [4–9]). Recently, this hypothesis received strong support from a series of reports that described attempts to selectively eradicate SCs in mice using genetic and pharmacological approaches [10–16]. In most models, a decrease in the number of cells with SC markers was associated with positive physiological outcomes that were interpreted as indications of rejuvenation. However, the accurate interpretation of these studies requires the reliable identification of SCs *in vivo*, and thus, depends on a level of exclusivity of SC markers that is currently lacking [17].

*In vivo*, SCs are conventionally identified by the presence of two markers extensively characterized *in vitro*: (i) elevated β-galactosidase activity and associated staining with X-Gal substrate detected at pH 6.0 (SAβG) [18] and (ii) expression of the *p16^Ink4a^* gene encoding a CDK4/6 inhibitor that contributes to the maintenance of proliferation arrest [19–22]. Notably, while these markers are attributed to mesenchymal SCs, there are a growing number of reports that several cell types express these traits as part of their normal physiology, independent of senescence (reviewed in [17]). We recently identified subtypes of macrophages that co-express *p16^Ink4a^* and SAβG [23]. Macrophages expressing these markers were elicited in young mice by SCs embedded in alginate beads (to prevent them from rapid eradication by immunocytes) and found to occur naturally within the adipose tissue of chronologically aged mice.

Macrophages are a critical component of innate and adaptive immunity, playing essential roles in the maintenance of tissue homeostasis [24,25]. Macro-phages are categorized by functional phenotypes associated with differential gene expression patterns. The best characterized phenotypes are of classical (M1) and alternative (M2) activation states, which reflect different physiological activities [26]. M1 polarization, which can be induced by LPS and type 1 cytokines (e.g. IFN-γ), is associated with pro-inflammatory responses to bacteria and viruses [26]. M2 polarization, which can be induced by type 2 cytokines (e.g. IL-4 and IL-13), is associated with anti-inflammatory response and regulation of wound healing [27]. Notably, macro-phages are characterized by a high phenotypic plasticity and exhibit a variety of mixed M1/M2 phenotypes allowing for rapid response and adaptation to a wide range of microenvironmental cues [28,29].

Macrophages have established roles in the pathogenesis of several age-associated diseases, including cancer [30,31], atherosclerosis [32,33], diet-induced obesity and insulin resistance [34–36], fibrosis [37–39] and osteoarthritis[40]; recently, SCs have been implicated in the same diseases [11,41–45]. The relative impact of *p16^Ink4a^*/SAβG-positive macrophages and SCs in age-related diseases is currently unclear, yet this understanding is crucial for the identification of therapeutic targets. Utilizing our previously described method for *in vivo* elicitation of *p16^Ink4a^*/SAβG-positive macrophages (intraperitoneal injection of alginate-encapsulated SCs [23]), we investigated the regulation of these markers in macrophages compared to mesenchymal SC. We identified several immuno-modulatory agents which reversibly up- or down-regulate *p16^Ink4a^* expression in macrophages, some of which demonstrated similar modulation of *p16^Ink4a^* expression in adipose tissue macrophages of chronologically aged mice. We found that the expression of *p16^Ink4a^* and SAβG in macrophages are markers of their physiological programs of polarization in response to immunomodulatory stimuli that are reversible and p53-independent, and therefore, clearly distinct from cellular senescence in which the expression of these biomarkers is constitutive following p53-dependent (at least in rodent cells) establishment of proliferation arrest. Taken together, these findings raise questions about the relative impact of specific subtypes of macrophages vis-à-vis SCs in driving the aging process and their potential role as cellular targets for anti-aging therapies.

## RESULTS

### Expression of *p16^Ink4a^* and β-galactosidase in macrophages is p53-independent

**Figure 1:**
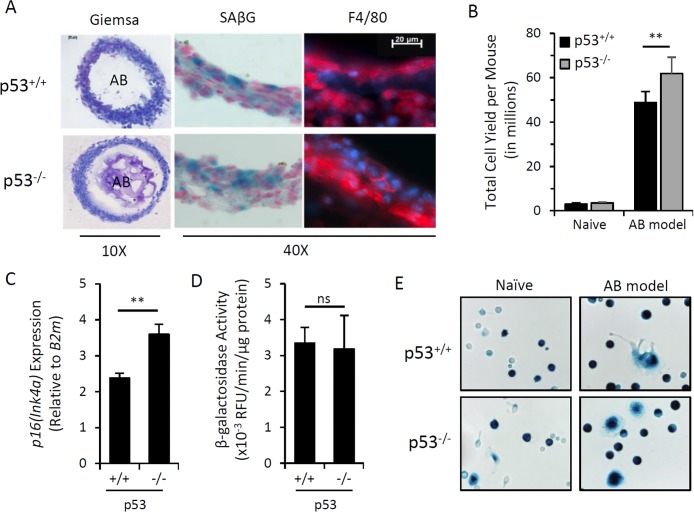
Induction of *p16^Ink4a^* and SAβG in macrophages does not require p53 Peritoneal lavage and alginate beads containing SCs (AB) were recovered from wild type (p53^+/+^) or p53 knockout (p53^−/−^) mice 15 days after injection (AB model). (**A**) Representative microphotographs of cryosectioned immunocyte capsules surrounding alginate beads stained with May-Grünwald-Giemsa for histology (10x objective), X-Gal substrate for β-galactosidase activity (SAβG; pH 6.0) (blue) with nuclear fast red counterstain (red), and an immunofluorescent antibody against macrophage marker F4/80 (red) with DAPI nuclear counterstain (blue) (40x objective). (**B**) Total yield of cells recovered from peritoneal lavage from naïve or AB-injected p53^+/+^ and p53^−/−^ mouse strains. (**C**) AB model-elicited immunocyte capsules were pooled equally from 3 mice and *p16^Ink4a^* gene expression relative to internal reference gene β2-microglobulin (*B2m*) was measured by qPCR. (**D**) β-galactosidase activity from cell extracts of immunocyte capsules from alginate beads recovered from individual mice was measured via 4-MUG hydrolysis, presented as the rate of 4-MU fluorescence (RFU) per minute normalized per microgram of protein. (**E**) Representative microphotograph of adherence-selected peritoneal lavage from naïve and AB-injected mice stained with X-Gal for SAβG activity. Data show mean ± standard deviation of two independent experiments (n=3 mice per experiment). Statistical comparison between p53^+/+^ and p53^−/−^ strains are indicated; ns, not significant; **, p-value < 0.01.

The tumor suppressor protein p53 (encoded by *Trp53*) is a key cell cycle regulator [46]. It has been widely reported that p53 is a crucial mediator of DNA damage-induced growth arrest and cellular senescence [46,47]. We utilized an *in vivo* model previously shown to generate *p16^Ink4a^*/SAβG-positive macrophages (i.e. intraperitoneal injection of alginate-encapsulated SCs) to evaluate whether *p16^Ink4a^*/SAβG-positive macro-phages could be elicited in p53-deficient mice, which are incapable of generating SCs. At two weeks post-transplantation, alginate beads were completely surrounded by layers of encapsulating immunocytes, exhibiting a similar morphology and abundance of macrophage marker F4/80 between p53-deficient and wild type mice (Figure 1A). The alginate bead model elicited a greater number of immunocytes into the peritoneal cavity of p53^−/−^ mice (Figure 1B). Together, these data suggest that p53 deficiency does not hinder immunocyte infiltration and general response to SC-containing alginate beads. We next examined whether p53 activity was required for the induction of *p16^Ink4a^* and SAβG in this model. Analysis of *p16^Ink4a^* mRNA via qPCR revealed increased expression in immunocyte capsules surrounding alginate beads in p53^−/−^ mice compared to wild type mice (Figure 1C). β-galacto-sidase activity evaluated via enzymatic 4-MUG hydrolysis and SAβG staining was unaffected by p53 deficiency (Figures 1A,D&E), consistent with previous reports of SAβG-positive macrophages in p53-deficient mice [48,49]. Thus, elevated *p16^Ink4a^* and SAβG expression in cells elicited by the alginate bead model is independent of p53 activity, and therefore, not a result of cellular senescence.

### *p16^Ink4a^*- and SAβG-positive macrophages remain responsive to polarizing stimuli

**Figure 2:**
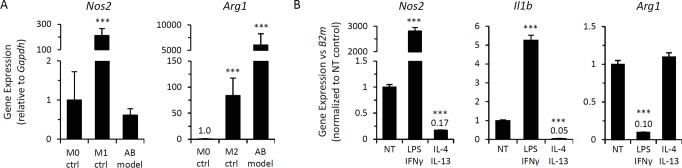
Macrophages elicited by alginate-encapsulated SCs possess a modulatable M2-like phenotype Gene expression analysis of macrophage polarization markers (M1, *Nos2* and *Il1b*; M2, *Arg1*) of alginate bead model (AB model)-elicited peritoneal macrophages from wild type mice via qPCR. (**A**) mRNA expression of *Nos2* and *Arg1* in AB-elicited macrophages adherence-selected from CD11b-enriched peritoneal lavage, as compared to expression in naïve bone marrow-derived macrophages (M0) or following polarization to M1 (IFN-γ for 24 hrs; M1 ctrl) or M2 (IL-4 for 24 hours; M2 ctrl) states. *Gapdh* expression was used an internal reference gene control. Data shows mean ± standard deviation (n=3). *** p-value < 0.001 compared to M0 control. (**B**) Peritoneal macrophages elicited by the alginate bead model were treated *ex vivo* with immunomodulatory agents. qPCR analysis of mRNA expression of indicated genes was normalized to β2-microglobulin (*B2m*) expression was determined following 72 hour incubation with M1-inducing stimuli (LPS at 1 ng/mL + IFN-γ at 10 ng/mL) or M2-inducing cytokines (IL-4 at 20 ng/mL + IL-13 at 10 ng/mL). Fold change in gene expression following treatment is depicted as mean ± standard deviation relative to non-treated controls; ***, p-value < 0.001. Results are representative of 3 independent experiments with peritoneal lavage cells pooled from at least 3 mice.

The polarization state of alginate bead model-elicited macrophages was evaluated via qPCR analysis of conventional M1 and M2 polarization markers (*Nos2* and *Arg1*, respectively). Expression profiles were compared to bone marrow-derived macrophages (BMDMs) stimulated with IFN-γ or IL-4 as controls for assessment of M1 and M2 polarization states, respectively. Alginate bead model-elicited macrophages enriched via adherence selection of CD11b-positive cells exhibited low *Nos2* expression compared to M1-polarized BMDMs (>500-fold lower), while *Arg1* expression was markedly elevated compared to M2-polarized BMDMs (>50-fold) (Figure 2A). The expression level of these polarization markers indicates that alginate bead model-elicited macrophages possess an M2-like phenotype.

Macrophages normally exhibit highly plastic pheno-types, demonstrating reversible polarization upon challenge with immunomodulatory stimuli. Therefore, we sought to determine whether *p16^Ink4a^*/SAβG-positive macrophages exhibit a reversible, physiologically responsive state, or if the co-expression of *p16^Ink4a^* and SAβG exists as part of a permanently acquired phenotype (such as a senescent or other refractory state [50]). To discriminate between these two possibilities, we first evaluated the responsiveness of M1/M2-associated gene expression profiles in alginate bead model-elicited macrophages following stimulation with M1- and M2-inducing agents (LPS/IFN-γ and IL-4/IL-13, respectively). LPS/IFN-γ induced high expression of *Nos2* (2,800-fold induction) and decreased expression of *Arg1* (10-fold) (Figure 2B), consistent with repolarization towards an M1 phenotype. In contrast, stimulation of these M2-like macrophages with IL-4/IL-13 resulted in a >5-fold decrease in *Nos2* expression, with elevated *Arg1* expression remaining unchanged, consistent with the maintenance of an M2-like state. Macrophage polarization affects the expression of cytokines that facilitate macrophage interactions with the microenvironment. Expression of M1-associated pro-inflammatory cytokine IL-1β increased following LPS/IFN-γ stimulation (5-fold) and decreased following stimulation with IL-4/IL-13 (>20-fold) (Figure 2B). Thus, M2-like *p16^Ink4a^*/SAβG-positive macrophages remain responsive to immunomodulatory stimuli, modulating gene expression in response to M1-inducing agents consistent with re-polarization.

### Reversible modulation of *p16^Ink4a^* and SAβG in response to macrophage polarization

**Figure 3:**
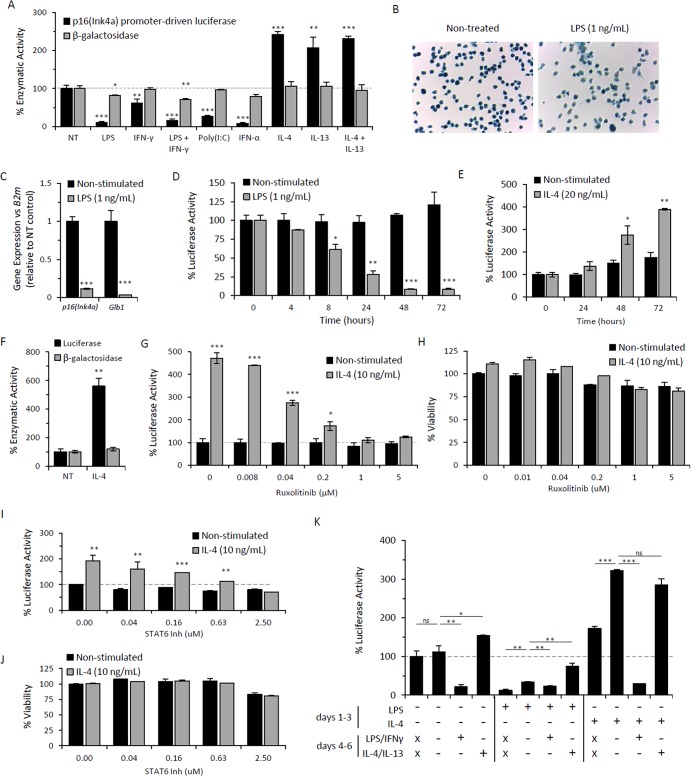
Immunomodulatory regulation of *p16^Ink4a^* and SAβG in macrophages Peritoneal lavage cells elicited by alginate-encapsulated SCs from *p16^Ink4a/Luc^* mice were treated *ex vivo* with immunomodulatory agents for 72 hours. (**A**) *p16^Ink4a^* promoter-driven luciferase activity (black bars) and β-galactosidase activity (via 4-MUG hydrolysis) (gray bars) were measured following treatment with M1- and M2-polarizing stimuli: LPS at 1 ng/mL, IFN-γ at 10 ng/mL, LPS/IFN-γ co-treatment, Poly(I:C) at 10 μg/mL, IFN-α at 100 U/mL, IL-4 at 20 ng/mL, IL-13 at 10 ng/ml, and IL-4/IL-13 co-treatment. Results are shown as the mean ± standard deviation for at least 3 independent experiments with statistical significance between treated and non-treated samples depicted. (**B**) Microphotograph of SAβG-stained adherence-selected macrophages with or without stimulation with LPS (1 ng/mL) for 72 hours (10x objective). (**C**) mRNA expression of *p16^Ink4a^* and β-galactosidase (*Glb1*) (relative to *B2m* expression) in macrophages from wild type mice with or without LPS stimulation for 72 hours analyzed via qPCR, as normalized to non-treated controls. Results depicted as mean ± standard deviation (n=3). (**D&E**) Kinetics of *p16^Ink4a^* promoter-driven luciferase activity per cell with or without LPS stimulation (**D**) or IL-4 stimulation (**E**), normalized to activity from non-treated cells at time zero. Results are shown as the mean ± standard deviation (n=3). Statistical significance with respect to non-treated control at time zero is indicated. (**F**) Luciferase activity and β-galactosidase activity (via 4-MUG hydrolysis) from proteose peptone-elicited lavage cells following stimulation with IL-4 (20 ng/mL) for 72 hours, normalized to non-treated controls. Results depicted as mean ± standard deviation (n=3). (**G-J**) Dose-dependent response of JAK1/2 inhibitor Ruxolitinib (**G&H**) and STAT6 inhibitor AS1517499 (**I&J**) on luciferase activity (**G&I**) and viability via CyQuant Direct assay (**H&J**) following 72 hours treatment of AB-elicited macrophages in the presence (gray bars) or absence (black bars) of IL-4 (10 ng/mL) stimulation. Results of luciferase activity and viability are representative of two independent experiments, depicted as mean ± standard deviation of data normalized to respective controls lacking inhibitors (with or without IL-4). Luciferase activity and viability are depicted as the percent signal relative to non-treated (NT) controls. Statistical significance between IL-4 stimulated and non-stimulated cells at each concentration of inhibitor is shown. Results are representative of three independent experiments, depicted as mean ± standard deviation. (**K**) Relative luciferase activity per cell following repolarization of AB-elicited macrophages (via adherence-enriched peritoneal lavage) with M1- and M2-inducing agents. Macrophages were left non-treated (NT) or treated with either LPS (1 ng/ml) or IL-4 (20 ng/ml) for 72 hours (days 1-3), as indicated. For each treatment set, samples were collected at 72 hours (no further treatment; days 4-6 = ×). Alternatively, cells were washed and placed in fresh medium (-), medium containing LPS (1 ng/mL) and IFN-γ (10 ng/mL), or medium containing IL-4 (20 ng/mL) and IL-13 (10 ng/mL) and incubated for an additional 72 hours prior to sample collection (as indicated for days 4-6). Luciferase activity is expressed as the percent activity per cell relative to non-treated (NT) controls after the first 72 hours. Results are representative of two independent experiments. *, p-value < 0.05; **, p-value < 0.01; ***, p-value < 0.001.

Studies utilizing *p16^Ink4a^*-deficient macrophages demonstrate a role for *p16^Ink4a^* in macrophage polarization [51]. However, the regulation of *p16^Ink4a^* expression in response to polarizing agents has not yet been described. To determine whether macrophage polarization regulates *p16^Ink4a^* and SAβG expression, peritoneal lavage cells from *p16^Ink4a/Luc^* mice elicited by the alginate bead model were stimulated with M1- and M2-inducing agents and subsequently, luciferase activity [driven by endogenous *p16^Ink4a^* promoter] and β-galactosidase activity via 4-MUG hydrolysis were measured (Figure 3A). Stimulation with the M1 polarizing TLR4 agonist LPS (1 ng/mL) decreased luciferase activity relative to non-treated control (80-90% decrease), with a statistically significant decrease in β-galactosidase activity (20-30% decrease) and decreased intensity of SAβG staining (Figure 3A&B). Consistently, expression analysis of *p16^Ink4a^* and β-galactosidase (*Glb1*) via qPCR confirmed substantial downregulation of both genes following LPS treatment of adherence-selected macrophages elicited via the alginate bead model in wild type mice (Figure 3C). The response of peritoneal lavage cells to LPS was rapid, with a significant decrease in luciferase activity observed within 8 hours (∼40% decrease) (Figure 3D). The co-treatment of LPS with IFN-γ, commonly used for M1 induction, showed similar effects to that of LPS alone (Figure 3A). Stimulation with additional M1-inducing agents, including type I and II interferons (IFN-α and IFN-γ, respectively) and Toll-like receptor 3 (TLR3) agonist Poly(I:C), decreased luciferase activity without affecting β-galactosidase activity (Figure 3A). These data demonstrate that M1-polarizing stimuli decreases *p16^Ink4a^* promoter activity and SAβG expression in macrophages.

In contrast to these observations, stimulation with M2-polarizing cytokines IL-4 and/or IL-13 increased *p16^Ink4a^* promoter-driven luciferase activity (2.1- to 2.4-fold) without modulating β-galactosidase activity (Figure 3A). IL-4-induced luciferase activity exhibited delayed kinetics (>48 hours) (Figure 3E). Similar findings were observed for peritoneal macrophages elicited by proteose peptone, indicating that the increased luciferase activity *in vitro* is not specific to alginate bead model-elicited macrophages (Figure 3F). Further, the IL-4-dependent increase in luciferase was abrogated by inhibitors of canonical IL-4-induced JAK-STAT signaling, including JAK1/2 inhibitor Ruxolitinib and STAT6 inhibitor AS1517499, without affecting viability (Figure 3G-J). No effect on *p16^Ink4a^* promoter activity was observed on macrophages treated with these inhibitors in the absence of IL-4, which, together with the delayed induction of p16Ink4a following IL-4 stimulation, suggest that *p16^Ink4a^* promoter induction occurs secondary to JAK-STAT signaling. Overall, these data demonstrate that *p16^Ink4a^* promoter activity is increased in response to M2-polarizing cytokines IL-4 and IL-13.

To further characterize the association of *p16^Ink4a^* expression with macrophage polarization, alginate bead model-elicited macrophages were polarized and subsequently challenged with stimuli to test the effects of repolarization on *p16^Ink4a^* expression (e.g. M1 to M2, or M2 to M1). We found that *p16^Ink4a^* promoter activity was reversibly modulated, showing elevated expression levels in response to M2 challenge and decreased expression levels in response to M1 challenge (Figure 3K). Thus, these data demonstrate that *p16^Ink4a^* is able to be reversibly modulated in macrophages by pathways associated with classical M1 and M2 polarization.

### Immunomodulatory regulation of *p16^Ink4a^* and SAβG is not an intrinsic property of senescent cells

**Figure 4:**
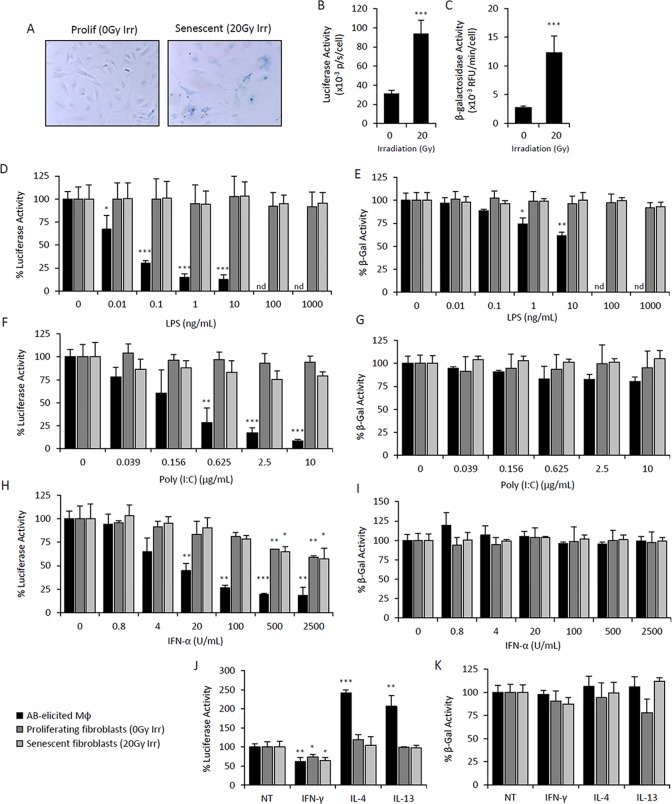
Elevated *p16^Ink4a^* and β-galactosidase is regulated by immunomodulatory agents in macrophages but not mesenchymal SCs Primary cultures of adipose-derived mesenchymal stromal cells (AdMSC) isolated from *p16^Ink4a/Luc^* mice were irradiated (20Gy) and cultured for 10 days for senescence induction. Mock irradiated cells were passaged and used as a proliferating cell control. Response of senescent and proliferating AdMSCs to immunomodulatory agents were compared to that of peritoneal lavage cells elicited by the alginate bead model. (**A-C**) Characterization of senescent and proliferating AdMSCs. Microphotographs of SAβG-stained cells depicts positive staining of senescent cells, as well as an enlarged and flattened morphology, compared to that of proliferating cell control (**A**). p16Ink4a promoter-driven luciferase activity (**B**) and β-galactosidase activity measured via 4-MUG hydrolysis (**C**) were measured in senescent and proliferating AdMSCs, confirming senescent phenotypes. (**D-K**) Dose-response curves of LPS (**D&E**), Poly(I:C) (**F&G**), IFN-α (**H&I**), and IFNγ (10 ng/mL), IL-4 (20 ng/mL) and IL-13 (10 ng/mL) (**J&K**) on *p16^Ink4a^* promoter-driven luciferase activity (left panels: **D,F,H&J**) and β-galactosidase activity measured via 4-MUG hydrolysis (right panels: E,G,I&K) after 72hr treatment. No effect on viability was observed via CyQuant Direct assay (>80% viability). Results are shown as the mean ± standard deviation for at least 3 experiments, with statistical comparison to non-treated controls; *, p-value < 0.05; **, p-value < 0.01; ***, p-value < 0.001. nd, not determined.

We next investigated whether the mechanisms regulating the activity of the *p16^Ink4a^* promoter and SAβG activity in macrophages were also present in SCs. Using cells isolated from p16*^Ink4a/Luc^* mice, the dose-dependent response of luciferase and β-galacto-sidase activities to immunomodulatory stimuli in macrophages was compared to that of proliferating and irradiation-induced senescent adipose-derived mesenchymal stromal cells. Mouse mesenchymal cells are capable of recognizing and responding to at least part of the immune stimuli tested (data not shown) [52]. Characterization of irradiated cells revealed phenotypes consistent with cellular senescence, including an enlarged morphology, positive SAβG staining, and increased *p16^Ink4a^* promoter activity and β-galactosidase activity (Figure 4A-C).

LPS stimulation reduced luciferase activity in alginate bead model-elicited macrophages, with maximal inhibition (∼85%) observed at concentrations ≥ 1 ng/mL (Figure 4D). In addition, β-galactosidase activity was reduced by 40% at 10 ng/mL (Figure 4E). In contrast, luciferase and β-galactosidase activities were unaltered in senescent and proliferating mesenchyme in response to LPS (up to 1 μg/mL) (Figure 4D&E). The effect of Poly(I:C) was also tested, revealing a dose-dependent decrease in luciferase activity that was selective to macrophages, while no effect was observed against β-galactosidase activity (Figure 4F&G). Treatment of macrophages with IFN-α resulted in stronger downregulation of *p16^Ink4a^* promoter activity and was effective at lower concentrations compared to mesenchymal cells (Figure 4H&I). Notably, the extent of IFN-α-induced suppression of luciferase activity observed in mesenchymal cells was independent of senescence. IL-4 and IL-13 stimulation exerted selective upregulation of luciferase activity in macro-phages (Figure 4J&K). IFN-γ stimulation decreased luciferase activity in macrophages and mesenchymal cells to a similar extent (30-40% inhibition) and independent of senescence (Figure 4J&K). Together, these data indicate that expression of *p16^Ink4a^* and SAβG in macrophages is regulated differently than in mesen-chymal cells in response to immunomodulatory stimuli.

### Poly(I:C) abrogates alginate bead model- and age-dependent increases in *p16^Ink4a^* expression *in vivo*

**Figure 5:**
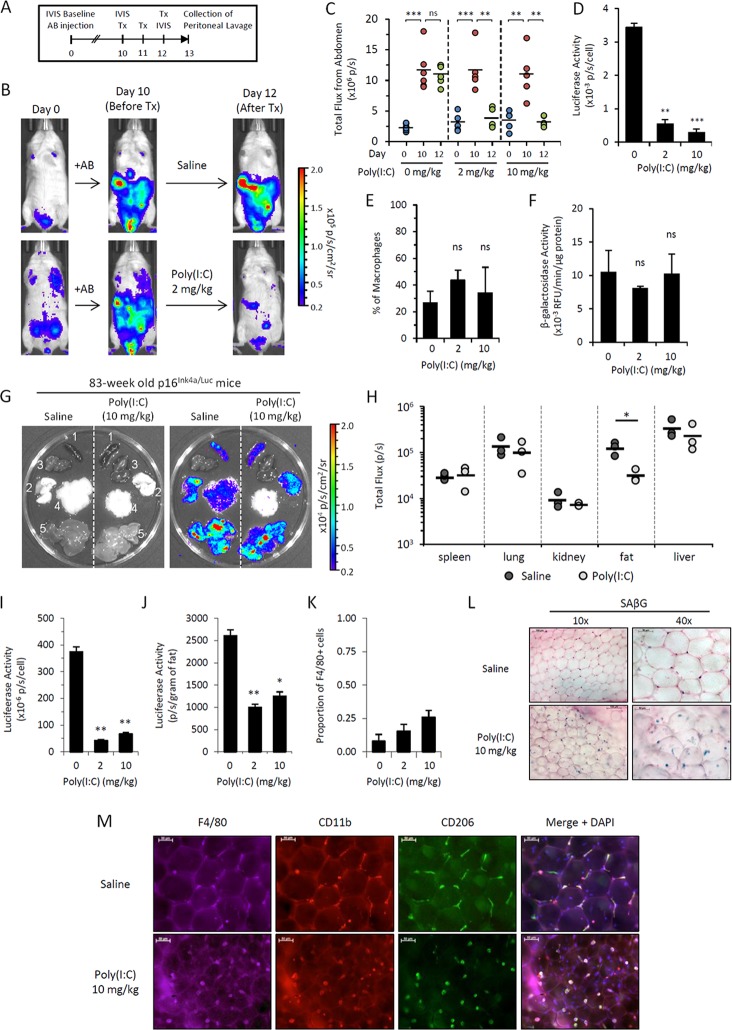
Poly(I:C) abrogates elevated *p16^Ink4a^* expression in two independent *in vivo* models (**A-F**) *p16^Ink4a/Luc^* mice injected with alginate-encapsulated cells (AB injection) were treated with Poly(I:C) in saline at 0, 2 and 10 mg/kg for 3 consecutive days. (**A**) Schematic representation of alginate bead model experiment depicting timeline and procedures. (**B**) Representative serial images of mice depicting bioluminescence before and after treatment with 2 mg/kg Poly(I:C). Colored scale depicts relative luminescent signal intensity (in radiance) of minimum and maximum thresholds, as indicated. (**C**) Graphical representation of bioluminescence (total flux; p/s) measured from the abdomen of treated mice on day 0 (prior to AB injection; blue), day 10 (after AB injection, prior to treatment; red), and day 12 (6 hours after the final treatment; green). Geometric mean is depicted. Statistical significant is calculated with respect to differences between indicated days within treatment groups. (**D-F**) The effects of Poly(I:C) treatment were analyzed in peritoneal lavage collected within 24 hours of the final treatment of 0, 2, and 10 mg/kg Poly(I:C). Luciferase activity (**D**), the proportion of peritoneal macrophages (CD45^+^ CD11b^+^ CD170^−^ F4/80^+^ cells) to total lavage cells as quantitated via flow cytometry (**E**) and β-galactosidase activity (measured via 4-MUG hydrolysis) (**F**) were quantitated from peritoneal lavage cells. Results are representative of two independent experiments (n=3-6 mice per group per experiment). Statistical significance compared to vehicle-treated controls is depicted; ns, not significant; **, p-value < 0.01; *** p-value < 0.001. (**G-M**) Chronologically aged mice (83-week old females) were treated with saline or Poly(I:C) (10 mg/kg) for 3 consecutive days. Organs were collected from mice the following day for quantitation of luciferase signal via IVIS. (**G**) Representative gray-scaled images of organs (1, spleen; 2, lungs; 3, kidneys; 4, perigonadal visceral fat; 5, liver) visualized on IVIS (left) with bioluminescence overlay in color (right). (**H**) Graphical representation of bioluminescence (total flux; p/s) quantitated from individual organs. Results are representative of two independent experiments (n=3 mice per group per experiment). *, p-value < 0.05. (**I-K**) Visceral perigonadal adipose tissue was pooled within groups, and the stromal vascular fraction was isolated for analysis. Luciferase activity per cell (I) was measured via luminometer, and the total signal per gram of fat (**J**) was calculated. (**K**) The proportion of cells in the SVF expressing macrophage marker F4/80 was measured by detection of immunofluorescent staining via cytometer. (**L&M**) Microphotographs of whole adipose tissue from mice with or without Poly(I:C) treatment (10 mg/kg) stained for SAβG activity via X-Gal reagent (blue; nuclear fast red counterstain) (**L**) and immunofluorescent staining of macrophage markers F4/80 (purple), CD11b (red), CD206 (green) and merged overlay with DAPI nuclear counterstain (blue) (**M**). Results are representative of two independent experiments.

Our data demonstrate that M1-polarizing stimuli downregulates *p16^Ink4a^* expression in macrophages *ex vivo*. We next investigated whether *p16^Ink4a^* expression was susceptible to M1-mediated regulation *in vivo* using the TLR3 ligand Poly(I:C). Upon intraperitoneal injection of alginate-encapsulated cells, *p16^Ink4a/Luc^* mice accumulate luciferase-positive macrophages that can be monitored *in vivo* via the IVIS imaging system (Figure 5A)[23]. Following induction of bioluminescence after 10 days post-alginate bead transplantation, mice treated intraperitoneally with Poly(I:C) (2 and 10 mg/kg) showed a complete abrogation of bioluminescent signal while the signal remained unchanged in vehicle-treated mice (Figure 5B&C). Analysis of peritoneal lavage cells from Poly(I:C)-treated mice revealed a >6-fold decrease in luciferase activity per cell compared to non-treated controls, with no difference in the proportion of CD11b^+^ F4/80^+^ macrophages (Figure 5D&E). Further, treatment with Poly(I:C) had no effect on β-galactosidase activity nor viability (>95%) of lavage cells, consistent with *in vitro* data (Figure 5F). Together, these data suggest that Poly(I:C) modulates *p16^Ink4a^* expression in macrophages *in vivo*.

We next investigated whether *p16^Ink4a^* promoter activity could be regulated by Poly(I:C) in aged mice, which have been shown increase *p16^Ink4a^*/SAβG co-expressing macrophages in their adipose tissues [23]. Chrono-logically aged mice (83-weeks old) were treated with Poly(I:C) and the luminescence of individual organs was quantitated via IVIS. Poly(I:C)-treated mice displayed a 4-fold reduction in luminescence from visceral adipose tissue, with no significant effects on other tissues, including spleen, lungs, kidney and liver (Figure 5G&H). Consistently, we observed a decrease in luciferase activity from the isolated stromal vascular fraction from Poly(I:C)-treated mice compared to saline-treated controls for signal normalized per cell or per gram of fat (Figure 5I&J). Staining of whole fat tissue revealed an increase in F4/80^+^ and SAβG^+^ cells in Poly(I:C)-treated mice (Figure 5L&M), consistent with a greater yield of F4/80^+^ cells quantitated from the stromal vascular fraction of cells via cytometric analysis (Figure 5K). Thus, the observed decrease in the luciferase signal in adipose tissue upon treatment with Poly(I:C) was not associated with macrophage depletion. These data suggest that *p16^Ink4a^* promoter activity in adipose tissue macrophages from aged mice is able to be modulated in response to immune stimuli.

## DISCUSSION

The accumulation of *p16^Ink4a^*-positive cells is observed in aged mice, and their eradication has been linked to certain improvements in the health state of older animals consistent with rejuvenation [10,11]. Even though *p16^Ink4a^*-positive cells *in vivo* have been assumed to be senescent, little evidence exists to directly support this assumption. Our previous work identifying macrophage subtypes that co-express markers conventionally assigned to SCs (*p16^Ink4a^*/SAβG) [23] has prompted additional interpretations of previously published experimental data regarding the role of *p16^Ink4a^*-positive cells in aging and age-related diseases, as recently articulated by Bennett and Clarke (2017) [54] and Kirkland and Tchkonia (2017) [55]. As such, defining the exact nature of *p16^Ink4a^*-positive cells is crucial for proper development of therapeutics for the prevention and treatment of aging and age-related diseases. Today, the field of aging is focused on the development of senolytic compounds that are isolated for their ability to selectively kill SCs generated *in vitro* [16,56,57]. If these cells are different from *p16^Ink4a^*-positive cells accumulating *in vivo* with age, this could misdirect both academic studies of senescence as a phenomenon, as well as practical efforts to develop anti-aging therapeutics. These considerations motivated our present work, which was aimed at defining the nature of *p16^Ink4a^*-positive cells found in mouse tissues *in vivo* and their relation to the phenomenon of cellular senescence.

What is “cellular senescence”? Currently, all definitions agree that SCs cease to proliferate. However, this parameter is not sufficient to define SCs since this is also the property of terminally differentiated cells. One apparent difference is that terminal differentiation occurs in response to various physiological stimuli, while induction of senescence almost always occurs in response to genotoxic stress (elevated ROS, radiation, replicative stress due to oncogene activation, telomeres fusion followed by DNA breaks, etc. [58]).

Accordingly, the onset of senescence commonly involves p53, a major universal genotoxic stress response mechanism that triggers cell cycle arrest, the first step in conversion to senescence [59]. Notably, rodent cells with no functional p53 fail to senesce and can be directly transformed into tumorigenic state by activated *Ras* [20]. Another intrinsic property of the senescent phenotype is that it is not reversible through known physiological stimuli, only occurring through the acquisition of genetic mutation or epigenetic modulations [60]. Thus, a more precise definition of SCs should include those cells that irreversibly cease to proliferate following genotoxic stress. Currently, none of the other properties of SCs that are being used for their recognition, such as *p16^Ink4a^*- or SAβG-positivity, are sufficiently specific for SCs as to be essential components of this definition [17].

We previously demonstrated that a significant proportion of *p16^Ink4a^*/SAβG-positive cells elicited by the alginate bead model, as well as those in the fat tissue of older mice, are of hematopoietic origin, express surface markers of macrophages and are capable of phagocytosis [23]. Here, we demonstrate that these cells appear and accumulate independently of their p53 status. Furthermore, induction of *p16^Ink4a^*/SAβG markers can be significantly modulated (in both directions) by physiological stimuli known to polarize macrophages. Notably, these stimuli failed to modulate expression of either marker in *bone fide* mesenchymal SCs. One such agent, Poly(I:C), demonstrated similar modulation of *p16^Ink4a^* within visceral adipose tissue of chronologically aged mice.

In recent literature, a role for *p16^Ink4a^* has been implicated in macrophage physiology with no relation to other properties of senescence. For example, *p16^Ink4a^* expression is induced during monocyte differentiation into macrophages *in vitro* without affecting the cell cycle, and macrophages from *p16^Ink4a^*-deficient mice are skewed towards an M2 phenotype, exhibiting defects in M1 polarization response [51]. Both findings are consistent with observations on human macrophages [61]. *p16^Ink4a^* was shown to suppress the secretion of IL-6 following LPS stimulation in macrophages, but not in rheumatoid synovial fibroblasts [62]. Therefore, our data demonstrating that *p16^Ink4a^* promoter activity can be modulated in macrophages in response to polarizing stimuli further support an emerging role for *p16^Ink4a^* in macrophages unrelated to cell cycle regulation. In fact, p53 and cyclin-dependent kinase (CDK) inhibitors (including *p16^Ink4a^*, *p19^Arf^* and *p21^Cip1/Waf1^*) have been implicated in the regulation of proinflammatory gene expression and secretion, as well as polarization, in macrophages [63–67]. Moreover, some of these processes are independent of CDK function and cell cycle regulation (reviewed in [68]), and thus, independent of their defined roles in SCs in the maintenance of permanent growth arrest.

In summary, we conclude that a significant proportion of *p16^Ink4a^*/SAβG-positive cells accumulating in aging mice are macrophages that acquired this phenotype as part of their physiological reprogramming towards an M2-like phenotype. This interpretation is consistent with reports that tumor-associated macrophages (TAMs), which possess also an M2 phenotype, were shown to express *p16^Ink4a^* [17,22]. Further, the tumor-promoting activities of TAMs can be reverted through polarization to M1 phenotype, e.g. by administration of Poly(I:C)[69,70], which we show is capable of decreasing *p16^Ink4a^* promoter activity *in vivo*.

It is highly unlikely that senolytic compounds isolated for their ability to eradicate *bona fide* SCs would be equally potent and selective against cells that simply resemble SCs by two unreliable biomarkers (*p16^Ink4a^*/SAβG) yet lack the most definitive properties of senescence. However, several molecules identified with anti-SC activities, including ruxolitinib [71], dasatinib [72–74] and quercetin [75–77], have documented anti-inflammatory effects on macrophages that may contribute to improvements in healthspan. We believe that the assumptions made in a series of recent works [10–16,43,45,78] - that *p16^Ink4a^*/SAβG-positive cells are SCs - needs to be carefully re-evaluated, and that the effects of anti-SC therapies on macrophages needs to be evaluated.

Importantly, our results do not overthrow the significance of the SC's role in aging or disprove the rationale for the development of senolytic compounds. Nevertheless, they do question the accuracy of interpretation of the reasons for the improvement of the health of mice following the eradication of *p16^Ink4a^*-positive cells, raising the possibility that SCs may not be the only ones implicated in age-related frailty and that other players may be involved that could require different approaches to target.

## MATERIALS AND METHODS

### Primary cell culture

Peritoneal lavage cells were isolated and cultured as previously described [23,53]. Briefly, peritoneal lavage was performed using saline supplemented with 2% heat-inactivated FBS (Gibco; Grand Island, NY), and cells were then pelleted via centrifuged and resuspended in DMEM/F12 (Life Technologies; Grand Island, NY) medium containing 10% heat-inactivated FBS, 100 units/mL of penicillin, 100 μg/mL of streptomycin and 2 mM L-glutamine. Peritoneal lavage cells from at least 3 mice were pooled for each experiment and cultured in a tissue culture incubator at 37°C and 5% CO_2_. The density of collected peritoneal lavage cells was measured using Via1-Cassette™, (ChemoMetec; Allerod, Denmark) where live and dead counts of nucleated cells were quantitated on a NucleoCounter® NC-200 (ChemoMetec) via staining with acridine orange and DAPI.

Primary human neonatal dermal fibroblasts (AllCells, LLC; Alameda, CA), pooled equally from three separate donors, were maintained in Dulbecco's modified Eagle Medium (DMEM) with phenol red supplemented with 10% (v/v) FBS, 100 units/mL of penicillin, 100 μg/mL of streptomycin and 2 mM L-glutamine, and 1X MEM non-essential amino acids. Cells were cultured in a tissue culture incubator at 37°C and 5% CO_2_.

Primary cultures of mouse adipose-derived mesenchymal stromal cells (AdMSC) were established from the stromal vascular fraction of peri-gonadal white adipose tissue from young *p16^Ink4a/Luc^* mice, as described [23]. Isolated adherent cultures were maintained in DMEM/F12 medium supplemented with 15% FBS and 1X anti-biotic/anti-mycotic solution (Thermo Fisher Scientific; Waltham, MA) in a tri-gas tissue culture incubator at 37°C, 5% CO_2_ and 3% O_2_. For induction of senescence, early passage cells were irradiated in suspension at 20 Gy, and replated cultures were main-tained at 21% O_2_ and 5% CO_2_ for at least 1 week prior to re-plating for treatment with immunomodulatory stimuli. Serial passaging of mesenchymal cells via enzymatic dissociation with TrypLE (Life Technolo-gies) was performed when confluency reached 80-90%.

### Animals

Albino C57BL/6J mice with hemizygous *p16^Ink4a^* knock-in of firefly luciferase (*p16^Ink4a/Luc^*) were obtained from our breeding colony, originally from Dr. Normal E. Sharpless [22]. C57BL/6J mice were obtained from Jackson Laboratories (Bar Harbor, ME). p53-knockout mice and age-matched C57BL/6 wild type controls, originally from Jackson Laboratories, were procured from our breeding colony. Animals were provided a commercial rodent diet (5% 7012 Teklad LM-485 Mouse/Rat Sterilized Diet, Harlan; Indianapolis, IN) and sterile drinking water ad libitum. All animals were confined to a limited access facility with environmental-ly-controlled housing conditions through-out the entire study period and maintained at 18-26°C, 30-70 % air humidity, 12-hour light/dark cycle. The animals were housed in micro-isolation cages under pathogen-free conditions, and if necessary, acclimatized in the housing conditions for at least 5 days prior to the start of the experiment. Animal usage in this experiment was approved under Institutional Animal Care and Use Committee (IACUC) at the Roswell Park Cancer Institute.

### Bioluminescence imaging

*In vivo* bioluminescence from the abdomen of mice was performed as previously described [23]. Briefly, mice were injected intraperitoneally with a 200 μl solution of 15 mg/mL D-luciferin potassium salt (Syd Labs; Boston, MA) in D-PBS without calcium and magnesium. At 10 minutes post-injection, isoflurane-anesthetized mice were placed into the IVIS Spectrum imaging system (PerkinElmer; Waltham, MA) for detection of luciferase activity (120-second exposure). Bioluminescence in *p16^Ink4a/Luc^* mice was quantified as total flux (p/s) of luminescent signal from the abdomen using via Living Image® software (PerkinElmer). For imaging of individual organs *ex vivo*, mice were perfused with 20 mL of D-luciferin in D-PBS (300 μg/mL). The organs were quickly excised, rinsed in D-PBS, and placed in the perfusion solution prior to positioning on a dry surface for IVIS imaging (120-second exposure).

### Alginate bead model

Preparation, implantation and subsequent retrieval of alginate beads containing irradiated human neonatal dermal fibroblasts (alginate bead model) into *p16^Ink4a/Luc^* (18-46 weeks old), p53-knockout (8-15 weeks old), and wildtype C57BL/6 mouse strains (8-15 weeks old), performed as previously described [23].

### *In vitro* treatments

Following elicitation of macrophages via the alginate bead model (2-3 weeks post-injection), or 1 mL of 3% proteose peptone (3 days post-injection) (BD Biosciences; Franklin Lakes, NJ), peritoneal lavage cells were collected and 2.5×10^5^ to 5.0×10^5^ cells per well were plated overnight in a 24-well plate in complete medium, followed by ≤ 72 hour treatments of carrier-free recombinant mouse cytokines IL-4, IL-13, IFNγ (PeproTech; Rocky Hill, NJ) and IFNα (BioLegend; San Diego, CA), LPS (Sigma Aldrich; St Louis, MO), high molecular weight (HMW) Poly(I:C) (InvivoGen; San Diego, CA), JAK1/2 inhibitor Ruxolitinib (ApexBio; Boston, MA) and STAT6 inhibitor AS1517499 (Axon Medchem; Reston, VA). Cell viability following treatment was assessed via metabolic-based resazurin viability assay and DNA-based CyQuant® Direct Cell Proliferation Assay (Thermo Fisher Scientific), with similar results.

For BMDM polarization phenotype controls, bone marrow cells of C57BL/6 were plated and stimulated with 30 ng/mL of M-CSF (eBioscience) for five days. On day 5, BMDMs were polarized with recombinant mouse cytokines, either 120 ng/mL IFNγ or 5 ng/mL IL-4 (R&D Systems, Minneapolis, MN), to obtain M1- and M2-polarized macrophages, respectively. M1- and M2-polarized BMDM RNA expression was compared to CD11b^+^ adherent cells from alginate bead elicited peritoneal lavage cells that were plated overnight in a 24-well plate in complete medium.

### *In vivo* Poly(I:C) treatment

*p16^Ink4a/Luc^* mice, either 10 days post-injection of alginate-encapsulated cells or naturally aged for 83-weeks, were injected intraperitoneally with up to 10 mg/kg of HMW Poly(I:C) in saline for 3 consecutive days. Bioluminescence from the abdomen *in vivo* and/or from organs *ex vivo* was measured via IVIS less than 24 hours after the last treatment.

### RNA analysis

Peritoneal lavage cells from mice injected with alginate-encapsulated cells (2 weeks post-injection) were adherence selected and then treated for up to 72 hours with M1- and M2-inducing stimuli. Total RNA was extracted with PureLink TM RNA Mini Kit (Thermo Fisher Scientific), quantified via spectrophotometer, and cDNA was synthetized using QuantiTech® Reverse Transcription kit (Qiagen; Hilden, Germany) from 0.5-1.0 μg of total RNA, according to the manufacturer's specifications. Quantitative PCR was performed using the ABI 7300 of Applied Biosystems (Foster City, CA). Probes for real-time PCR were purchased from Integrated DNA Tehcnologies (IDT; Coralville, IA). At the 5′ end, probes were conjugated to the fluorochrome FAM with internal quencher ZEN and at the 3′ end the quencher IovaBlack® FQ. The internal reference gene control *B2m* was designed with TAMRA at 5′ end. The cycle conditions for quantitative real-time PCR using PrimeTime® Gene Expression Master Mix (IDT) were 2 minutes of uracil-DNA glycosylase pre-treatment at 50°C and 95°C for 15 min, followed by 40 cycles of 94°C for 1 min, and 60°C for 1 min. Gene-specific primers and probe sets were purchased from IDT for *Nos2* (Mm.PT.58.43705194), *Arg1* (Mm.PT.58.8651372), *Glb1* (Mm.PT.58.8893651), *Il1b* (Mm.PT.58.41616450), *p16^Ink4a^* (Mm.PT.58.42804808). Characterization of BMDM polarization controls together with alginate bead-elicited peritoneal macrophages were performed using quantitative RT-PCR with SYBRgreen detection (BioRad), using the following primer sets (IDT): *Gapdh*, Forward 5′-ggcaagttcaacggcacagtcaag-3′, Reverse 5′-gcacatactca gcaccagcatcac-3′; *Nos2*, Forward 5′-acaagctgcatgtga catcg-3′, Reverse 5′-ggcaaagatgagctcatcca-3′; *Arg1*, Forward 5′-AAGAAAAGGCCGATTCACCT-3′, Reverse 5′-CATGATATCTAGTCCTGAAAGG-3′.

### Quantitation of macrophages

Quantitation of macrophages (CD11b^+^ F4/80^+^ CD170^−^ cells) from peritoneal lavage was performed via flow cytometry, as described [23]. Alternatively, 1.5×10^5^ cells were incubated in Fc blocker solution (1:100 dilution of anti-CD16/32 in D-PBS supplemented with 2% heat-inactivated FBS and 1mM EDTA) for 10 minutes at room temperature, followed by a 30 minute incubation with anti-CD11b (AlexaFluor488-conjugated) and anti-F4/80 (AlexaFluor647-conjugated) antibodies (1:50 final dilution). Afterwards, cells were washed 3 times, resuspended D-PBS supplemented with 2% heat-inactivated FBS, 1mM EDTA and 10μg/ml Hoechst-33343 and incubated at 37°C for 15 minutes prior to analysis via the NC-3000 cytometer (ChemoMetec) using the 3-color acquisition protocol according to manufacturer instructions. Data analysis was performed using NC-3000 analysis software. All antibodies were obtained from BioLegend.

### Enzymatic assays for luciferase and β-galactosidase activity

Cell lysates were prepared in 1X Reporter Lysis Buffer (Promega; Madison, WI) supplemented with 0.5% Triton X-100 (Sigma). Luciferase activity of cell lysates was assessed using Bright-Glo™ Luciferase Assay System (Promega) according to manufacturer's instructions. β-galactosidase activity of cell lysates was assessed as previously described [23] with minor modifications. Briefly, kinetics of enzymatic hydrolysis of 4-methylumbelliferyl-β-D-galactopyranoside (4-MUG) (Invitrogen) was determined in citrate-phosphate buffer at optimal acidic β-galactosidase reaction pH (4.0) at room temperature by measuring fluorescence (Ex/Em 360nm/440nm) at regular intervals over 1 hour. Activities were normalized per cell number or per amount of protein measured by the Pierce BCA Protein Assay Kit (Thermo Fisher Scientific) per the manufacturer's instructions. The Infinite® M1000 PRO microplate reader (Tecan; Männedorf, Switzerland) was utilized for data acquisition of all assays.

### Tissue staining

Immunofluorescence staining of whole perigonadal adipose tissue was performed as previously described [23]. Briefly, thin samples (2-3 mm) of adipose tissue were fixed 4 hours in 4% formaldehyde in PBS at 4°C, washed in PBS overnight, incubated with block solution (PBS with 5% normal rat serum and 0.25% triton x-100) 1 hour at room temperature and stained with a cocktail of rat monoclonal antibodies against F4/80 (AlexaFluor647-conjugated), CD11b (AlexaFLuor594-conjugated) and CD206 (AlexaFluor488-conjugated) 4 hours at room temperature. Antibodies were diluted 1:50 in blocking solution. Nuclei were counterstained with DAPI (Invitrogen). Samples were washed 1 hour with PBS, cleared and mounted in glycerol.

SAβG staining of glutaraldehyde-fixed whole adipose tissue was performed as previously described [23]. Tissues were stained for less than 12 hours at 37°C, counterstained with nuclear fast red, cleared and mounted in glycerol.

For histology, SAβG staining and immunohisto-chemistry on sections, samples were fresh-frozen in Neg-50 freezing medium (Thermo Fisher Scientific). Next, 12-μm sections were prepared on a cryotome CM1900 (Leica Biosystems; Wetzlar, Germany). SAβG-stained sections were counterstained with nuclear fast red, dehydrated, cleared in xylene and mounted with DPX. For F4/80 macrophage marker visualization, sections were fixed with 4% formaldehyde in PBS for 5 minutes at room temperature and washed three times with PBS. Sections were incubated with blocking solution 15 minutes at room temperature and stained with AlexaFluor594-conjugated rat monoclonal antibody against F4/80 (diluted 1:50 in blocking solution) for 30 minutes at room temperature. After washing with PBS, sections were mounted with ProLong Diamond anti-fade reagent with DAPI. For morphological analysis, unfixed sections were stained by May-Grünvald method.

All images were acquired with AxioImager Z1 (Carl Zeiss Inc.; Oberkochen, Germany) microscope (bright field for morphology and SAβG; epifluorescence for F4/80 and DAPI staining) using AxioVision software (Zeiss). All antibodies were obtained from BioLegend.

### Statistical analysis

All data are presented as means ± standard deviation. For *in vitro* studies, each experiment consists of cells pooled from at least 3 animals and assayed in duplicate. Experiments were repeated at least three times with cells isolated and pooled from different mice, with similar results were obtained across experiments. Statistical comparison of two groups was performed using an unpaired Students' two-tailed t-test. Differences were considered statistically significant at p-values less than 0.05: not significant (ns; P > 0.05), P > 0.05; *, P ≤ 0.05; **, P ≤ 0.01; ***, P ≤ 0.001. All statistical analyses were performed using GraphPad Prism version 5.00 (GraphPad Software, San Diego, CA).

## Abbreviations

AdMSC: mouse adipose-derived mesenchymal stromal cells
BMDM: bone marrow-derived macrophages
IFN: interferon
LPS: lipopolysaccharide
Poly(I:C): polyinosinic:polycytidylic acid
qPCR: quantitative real-time polymerase chain reaction
SAβG: senescence-associated β-galactosidase
SASP: senescence-associated secretory phenotype
SCs: senescent cells
